# THE BIDIRECTIONAL RELATIONSHIP BETWEEN AGING IN PLACE AND COGNITIVE FUNCTIONING OVER TIME

**DOI:** 10.1093/geroni/igz038.1543

**Published:** 2019-11-08

**Authors:** Jonathan Platt, Yvonne Michael, Gina Lovasi, Andrea Rosso

**Affiliations:** 1 Columbia University, New York, New York, United States; 2 Drexel University, Philadelphia, Pennsylvania, United States; 3 Pittsburgh School of public health, pittsburgh, Pennsylvania, United States

## Abstract

Residential stability (aging in place) in older adults may be either supportive or detrimental to cognitive aging, and may be dynamic over time. Using residential histories of 3608 older adults in the Cardiovascular Health Study, this study seeks to estimate the potentially bidirectional relationship between residential change and cognitive functioning. Residential data were recorded and georeferenced annually, and the Modified Mini-Mental State Examination assessed global cognitive functioning. Marginal structural models will be used to assess the effect of residential and cognitive exposures over time, in the presence of time-varying covariates that may act as confounders and mediators at different time points. We hypothesize that residential stability will have a bidirectional relationship with cognitive functioning over time. Aging in place will be associated with higher cognitive function during follow-up, and predict longer dementia-free survival. In turn, time to residential relocation during follow-up will be shorter among those with lower cognitive function.

